# Missing by omission: the hidden heart of cardiovascular inequity in the UK

**DOI:** 10.3399/BJGP.2025.0701

**Published:** 2026-05-01

**Authors:** Clare R Goyder, Caroline Mitchell, Charlotte James, Oshin Sharma, Ben Lacey, Nigel David Hart, Emily McFadden, Joseph J Lee, Yeyenta Osasu, Pensee Wu, Kathryn S Taylor, John Ford

**Affiliations:** 1 Nuffield Department of Primary Care Health Sciences, University of Oxford, Oxford, UK; 2 School of Medicine, Keele University, Keele, UK; 3 Population Health Sciences, Bristol Medical School, University of Bristol, Bristol, UK; 4 Population Health Sciences Institute, Newcastle University, Newcastle upon Tyne, UK; 5 Associate Professor of Medicine and Epidemiology, University of Oxford, Oxford, UK; 6 Honorary Consultant Physician in Public Health, Oxford University Hospitals NHS Foundation Trust, Oxford, UK; 7 School of Medicine, Dentistry & Biomedical Science, Queen’s University Belfast, Belfast, UK; 8 University of Sheffield, Sheffield, UK; 9 Wolfson Institute of Population Health, Queen Mary University of London, London, UK

## Missingness as a driver of persistent disparities in a universal healthcare system

Cardiovascular disease (CVD) remains a leading cause of premature mortality in the UK contributing to a quarter of all deaths.^1^ It is one of the largest drivers of health inequalities nationally, accounting for over one-fifth of the life expectancy gap between most and least deprived communities in males and females.^2,3^ Data from the Office for Health Improvement and Disparities from 2022–2023 show that in males there were 15 935 excess deaths and in females there were 9707 excess deaths attributable to circulatory disorders in people living in the most socioeconomically deprived quintile when compared to the least deprived quintile (Figure 1 and Figure 2).^3^ With £50 million investment, the National Institute for Health and Care Research (NIHR) is establishing new research consortia to tackle these stark disparities in cardiovascular health, particularly in higher risk groups such as ethnic minorities and deprived communities; and seeks to address inequalities in CVD outcomes in women and men.^4^

**Figure 1.**
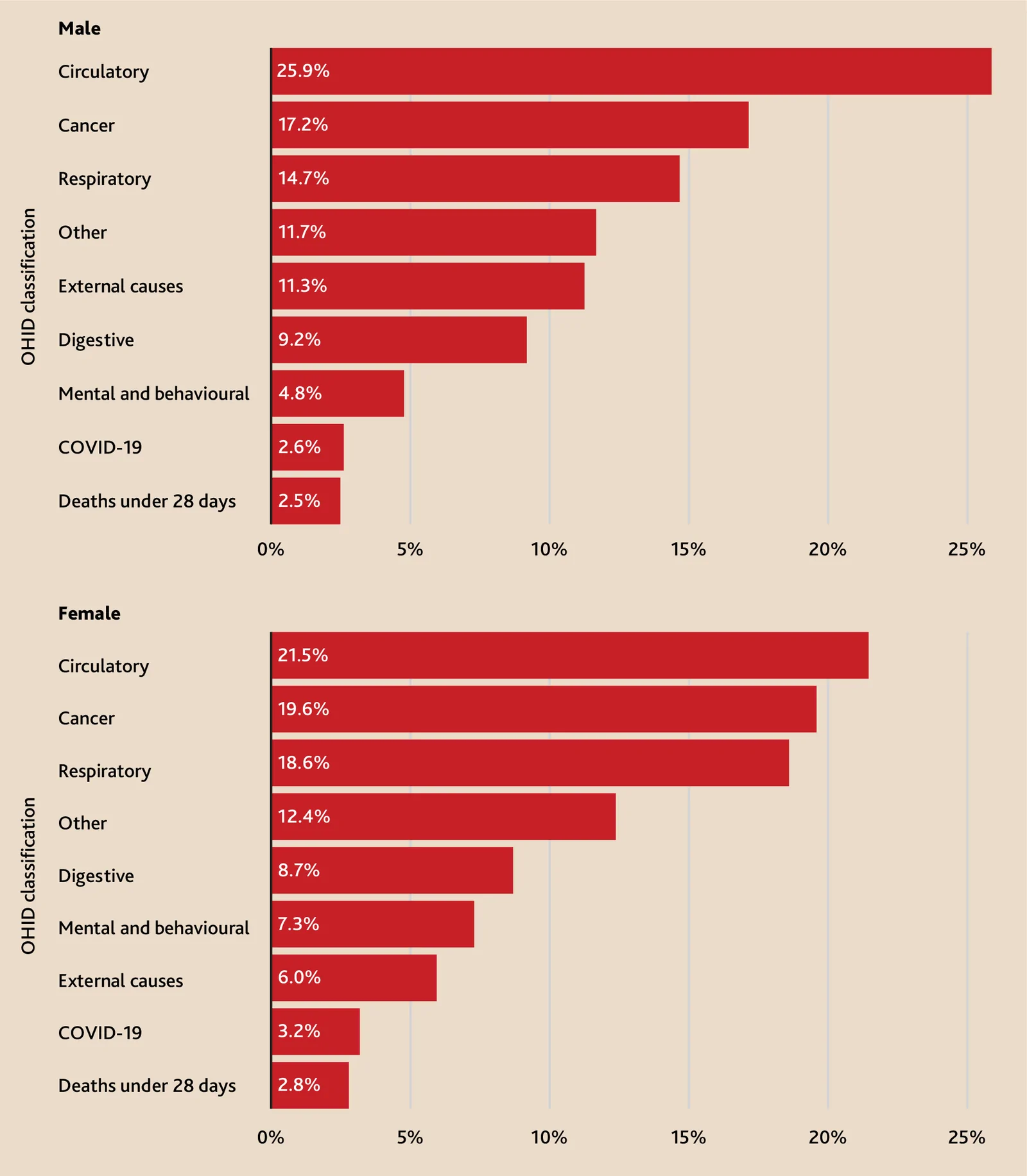
Breakdown of the life expectancy gap between the most and least deprived quintiles of England by cause of death (2022 to 2023). Group definitions follow OHID classifications: circulatory includes heart disease and stroke; respiratory includes flu, pneumonia, and chronic lower respiratory disease; digestive includes alcohol-related conditions such as chronic liver disease and cirrhosis; external includes deaths from injury, poisoning, and suicide; and mental and behavioural includes dementia and Alzheimer’s disease. Data from OHID, Segment Tool;^3^ based on Office for National Statistics death registration data, 2021 Census-based mid-year population estimates, and Ministry of Housing, Communities and Local Government Index of Multiple Deprivation, 2019. Created with Datawrapper. OHID = Office for Health Improvement and Disparities.

**Figure 2.**
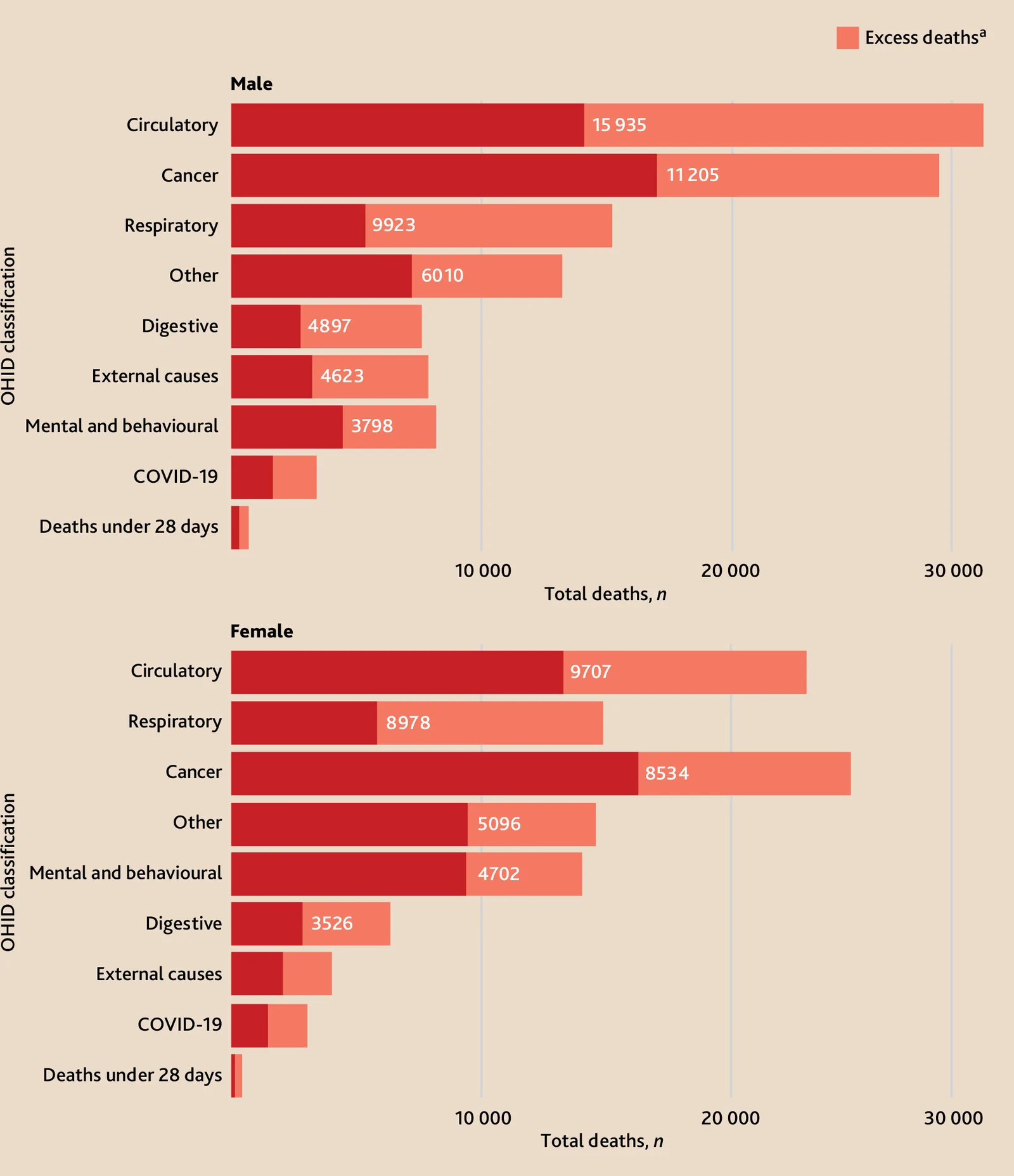
Breakdown of total and excess deaths in the most deprived quintile of England by cause of death (2022 to 2023). Group definitions follow OHID classifications: circulatory includes heart disease and stroke; respiratory includes flu, pneumonia, and chronic lower respiratory disease; digestive includes alcohol-related conditions such as chronic liver disease and cirrhosis; external includes deaths from injury, poisoning, and suicide; and mental and behavioural includes dementia and Alzheimer’s disease. ^a^Excess deaths are the number of deaths that would be avoided in the most deprived quintile if it had the same mortality rate as the least deprived quintile. Data from OHID, Segment Tool;^3^ based on Office for National Statistics death registration data, 2021 Census-based mid-year population estimates and Ministry of Housing, Communities and Local Government Index of Multiple Deprivation, 2019. Created with Datawrapper. OHID = Office for Health Improvement and Disparities.

Here we highlight that to close the gap in cardiovascular outcomes, researchers, policymakers, and practitioners must address the issue of *missingness*. The concept of missingness was given prominence by Williamson in regard to missed clinical appointments, particularly in inclusion health groups.^5^ However, missingness cuts across much of cardiovascular inequity: the people missing out entirely on opportunities to prevent CVD because they never have risk factors diagnosed or treated; the communities invisible in financial incentives, capitation formulas, and in health research. Missingness shapes and amplifies the apparent evidence base, and not addressing it risks embedding inequity further into the fabric of data-driven policy. Missingness is also structural and systemic. People are not only missed by chance or purposely omitted, they are overlooked from the outset because data-driven health systems are not designed to measure those who are missing.^6^

## Missingness in diagnosis, treatment, and appointments

Many people, particularly in inclusion health groups and those living in disadvantaged areas, remain underdiagnosed and undertreated for the ‘ABCV’ of CVD prevention: atrial fibrillation, high blood pressure (hypertension), raised cholesterol, and vaccination.^7^ While the triad of ABC is well-established, the European Society of Cardiology now includes adult vaccination for respiratory infections as a proven and underused foundational pillar.^8^ This makes achieving immunisation equity even more crucial, especially as vaccination uptake is lower in deprived areas.^9^ Missingness in diagnosis and treatment, and variation in vaccination uptake may also be explained by missingness in appointments, where there is also a clear deprivation gradient.^5^ Digital exclusion and poor digital health literacy further compound missingness in health care and make care even less accessible. These commonly impact older adults, individuals with lower educational attainment, and those with limited English proficiency, and are associated with broader structural and intersectional disadvantage. These disparities are further exacerbated by variations in levels of trust in digital tools and variation in their uptake, strongly influenced by age, socioeconomic status, and ethnicity.^10^

One of the first documented examples of strategies to mitigate for missingness in diagnosis and treatment showcased the importance of outreach in cardiovascular prevention and came from the work of GP, Julian Tudor Hart. Many inequalities result from decades of mismatch between health need, policy, and research inclusion, and this is a far cry from the solidarity shown by Hart’s pioneering work and the communities in the Welsh Valleys during the interwar years, which laid the foundations for our NHS. Hart defined the *‘inverse care law’,* describing how people who need health care the most are the least likely to receive it, and this underpinned his approach to general practice.^11^ At their Glyncorrwg practice, Hart and his wife, Mary (a researcher and practice nurse), challenged the normalisation of premature cardiovascular death in disadvantaged communities by embedding research-informed, data-driven, proactive initiatives in community engagement and prevention into routine care. His 1982 article, *Measurement of omission*, argued that health systems only measure what they do and fail to measure what they do not do, and that GPs with registered populations should focus on this gap. Mary and Julian Hart measured the blood pressure of every adult in Glyncorrwg, a first in the UK.^12^ The inclusion of blood pressure measurement for the complete adult practice population improved CVD outcomes for the whole community; indeed after 25 years, premature mortality was 30% lower in Glyncorrwg than in neighbouring villages.^13^

## Missingness by financial incentives and capitation formula

Missingness in diagnosis and treatment is exacerbated by missingness in the financial structures of general practice. The Quality and Outcomes Framework (QOF) was designed to improve and standardise primary care but failed to include everyone, in stark contrast to Hart’s inclusive approach. QOF financially incentivises general practices to achieve a specified threshold of quality indicators within their population. Personalised Care Adjustments, also known as exception reporting, allows practitioners to exclude patients from quality indicators. However, more patients are excluded in areas of greater deprivation. The combination of thresholds and ability to exclude patients increase the propensity for recurrent missingness.^14^ QOF has been maintained in England for two decades; there is no evidence that its replacements in the devolved administrations have fared any better in respect of missingness.

Increasing workload and decreasing finance in general practice are the main drivers of continuing inequity of provision. Moreover, these insufficient and declining resources are inequitably distributed in relation to need. There have been very limited moves to match health needs with primary care services, which is fundamental to mitigating the inverse care law.^11^ The Carr–Hill formula is a UK-wide capitation formula for general practices. It is a contract reimbursement formula to allocate funding for the services practices provide. It is primarily a workload formula, reflecting the work undertaken by GPs to provide patients with access to general medical services, with adjustment for need factors.^15^ This approach does not account for missingness but rather provides more funding to practices that already have a high workload due to patient demand. Practices that have a large proportion of missing patients, who do not regularly attend and who are more likely to be underdiagnosed, are given less funding.^15^ The Health Foundation and Nuffield Trust urge replacing the Carr–Hill formula with needs-based funding that reflects deprivation and health inequalities, using models like integrated care board allocation or targeted initiatives, such as enhanced primary care network-based case finding and intervention.^16^

## Structural missingness in research and health data

Those most underserved by prevention and treatment of CVD are also often under-represented in research. Evidence-*biased* medicine prevails, with populations of most cardiovascular clinical trials (including on statins) characterised by representation bias towards white, affluent, healthier, more health literate male participants.^17^ Many studies also exclude those with multiple long-term conditions and pregnant women. This mismatch between research design, delivery, and real-world need may also contribute to stalled or declining healthy life expectancy in high-risk and underserved groups, and perpetuates a cycle of exclusion from evidence-informed cardiovascular care.

Under-representation of high-risk patients in research extends beyond clinical trials. Population-wide electronic health records (EHRs), collected as part of routine care, can now be securely accessed for population health management and research. But disparities in diagnosis, treatment, reporting, and access translate to structural missingness in EHR data. If not addressed, these disparities will introduce bias. A prime example of this is in algorithm development, including artificial intelligence (AI).^18^ If structural missingness is not measured and accounted for, data-driven cardiovascular research risks widening existing inequalities. To mitigate for this, ‘data disaggregation’ — reporting of research outcomes in population sub-groups with intersecting sociodemographic, geographical, and health risk factors — will be key. Moreover, when missingness is not random there will be spurious associations in the dataset that are not found in the general population. When developing predictive algorithms, using either AI or more ‘traditional’ methods, there needs to be a shift from relying solely on evaluation metrics that measure performance, to including metrics that measure fairness. There are necessary trade-offs between competing concepts of fairness, but this should be acknowledged and measured in research. Achieving true ‘predictive parity’, or other measures of fairness, to ensure algorithms do not widen or perpetuate inequalities, will require accounting for the structural missingness present in EHR data.

## Conclusion

Inequalities in cardiovascular diagnosis and management in the UK persist despite state-funded universal health care, with underserved populations continuing to be missing by omission. Missingness prevails in prevention, diagnosis, treatment, and appointment attendance. This is further compounded by structural missingness within research and health-related data collection, and entrenched missingness within financial incentives and funding formulae. But missingness is not inevitable. It is possible to improve engagement of underserved groups in both research and clinical practice. The NIHR’s new inclusion strategy now explicitly embeds inclusion in all stages of research and impact but the effects of this will only be seen in time. This fundamental shift is much needed and should be guided by the principle of proportionate universalism as Sir Michael Marmot advised:

*‘Actions should be universal, but with an intensity and a scale proportional to the level of disadvantage*.’^19^

Moreover, across the country there are many examples where locally recruited and trained community health and wellbeing workers play a pivotal role in supporting underserved communities to access health care.^20^

The NIHR CVD investment offers a timely opportunity to reimagine cardiovascular health equity and could truly be the catalyst for transformative change; but its success will depend on sustained commitment to systemic reform, genuine engagement with public, patients, and frontline practitioners, and, above all, a determination to both measure, characterise, and confront missingness, in all its forms. If research questions and methods are shaped by data availability rather than unmet need, there is a risk of perpetuating the invisibility and silence even further, especially for those who are already unseen and unheard. Julian and Mary Hart demonstrated that *‘whole population care helps reduce mortalit*y*’*; their inclusive approach inspires a collective responsibility to ensure that no one is missing by omission, and they leave an enduring legacy and philosophical framework that should underpin both CVD prevention and health equity research in primary care.^12,13^
